# Reviewed article: Paiva RFPS, Maia ALO, Martins JBC. Occurrences, human harm and years of life lost due to natural disasters in the state of Rio de Janeiro, Brazil, 2010-2022: a cohort study. Epidemiol Serv Saude. 2025;34:e20240412. 10.1590/S2237-96222025v34e20240412.en

**DOI:** 10.1590/S2237-96222025v34e20240412.a

**Published:** 2025-04-18

**Authors:** Oswaldo Villa-Campos

**Affiliations:** 1 Universidade de São Paulo, São Paulo, SP, Brasil

## Peer review A

## Questionnaire

Does the manuscript contain new and significant information to justify publication? No

Does the Abstract (Summary) clearly and accurately describe the content of the article? Yes

Is the problem significant and concisely stated? No

Are the methods described comprehensively? No

Are the interpretations and conclusions justified by the results? Yes

Is adequate reference made to other work in the field? No

## Manuscript Structure

Length of article is: Adequate

Number of tables is: Adequate

Number of figures is: Adequate

Please state any conflict(s) of interest that you have in relation to the review of this paper (state “none” if this is not applicable).

None

Interest: Below

Quality: Below

Originality: Average

Overall: Average

Recommendation: Minor Revision

## Comments

A pergunta de pesquisa precisa ser mais bem definida. “Considere usar a estrutura população, intervenção exposição, comparador e desfecho na definição da pergunta de pesquisa”. (PICO).” “Esclareça a hipótese e como ela foi testada.”

A formatação do MÉTODOS não tem raciocínio precisa descrever corretamente como os guidelines indicam “Descreva os métodos com mais detalhes, incluindo o processo de coleta e análise de dados, para que outros pesquisadores possam replicar o estudo.” “Explique como os vieses foram minimizados e a validade interna foi garantida.” nos métodos tem um trecho que aparentemente podária ser parte da introdução.

“As tabelas e figuras precisam ser revisadas para garantir clareza, precisão e evitar redundância com o texto.”

“As figuras devem ser autoexplicativas.” “as tabelas não são claras faltam dados e descrição mas detalhada por exemplo (media,, DP, não fica claro a leitura das tabelas) 

“Verifique a pertinência de incluir as seguintes palavras-chaves já existentes no DeCS e que melhor descrevem sua pesquisa:

“O texto precisa ser revisado para garantir clareza, precisão e objetividade na linguagem.” “As ideias devem ser apresentadas de forma organizada e lógica.” “seguindo uma linha de raciocínio claro com continuidade”.

“Revise a lista de referências, verificando se inclui os trabalhos mais relevantes e recentes sobre o tema.” “Inclua referências importantes que podem ter sido omitidas.” “referencias de sites de internet poderiam no ter o mesmo peso que um artigo cientifico”

“Assegure-se de que as ilustrações contribuam para a clareza e o impacto visual do trabalho.” verifique que o tamanho das figuras estejam acordo com a guia da revista”

